# PIN2 Turnover in Arabidopsis Root Epidermal Cells Explored by the Photoconvertible Protein Dendra2

**DOI:** 10.1371/journal.pone.0061403

**Published:** 2013-04-18

**Authors:** Ján Jásik, Barbara Boggetti, František Baluška, Dieter Volkmann, Thomas Gensch, Twan Rutten, Thomas Altmann, Elmon Schmelzer

**Affiliations:** 1 Max Planck Institute for Plant Breeding Research, Köln, Germany; 2 Department of Dermatology, Center for Molecular Medicine Cologne, Köln, Germany; 3 Institute of Cellular and Molecular Botany, University of Bonn, Bonn, Germany; 4 Institute of Complex Systems 4 (ICS-4, Cellular Biophysics), Researche Centre Juelich, Juelich, Germany; 5 The Leibniz Institute of Plant Genetics and Crop Plant Research, Gatersleben, Germany; 6 Faculty of Natural Sciences, Comenius University, Mlynská dolina, Bratislava, Slovakia; Centro de Investigación y de Estudios Avanzados del IPN, Mexico

## Abstract

The steady state level of integral membrane proteins is dependent on a strictly controlled delivery and removal. Here we show that Dendra2, a green-to-red photoconvertible fluorescent protein, is a suitable tool to study protein turnover in plants. We characterized the fluorescence properties of Dendra2 expressed either as a free protein or as a tag in Arabidopsis thaliana roots and optimized photoconversion settings to study protein turnover. Dendra2 was fused to the PIN2 protein, an auxin transporter in the root tip, and by time-lapse imaging and assessment of red and green signal intensities in the membrane after photoconversion we quantified directly and simultaneously the rate of PIN2 delivery of the newly synthesized protein into the plasma membrane as well as the disappearance of the protein from the plasma membrane due to degradation. Additionally we have verified several factors which are expected to affect PIN2 protein turnover and therefore potentially regulate root growth.

## Introduction

The plasma membrane is a highly dynamic structure whose components undergo continuous modification and renewal. Proteins turnover in the membrane, i.e. their internalization by endocytosis and delivery by exocytosis, is strictly regulated to respond adequately to environmental and developmental cues. Internalized proteins may be sorted for gradual degradation and some portions may be recycled back to the plasma membrane [1], [2]. Although protein abundance can be easily evaluated by classical approaches such as western blotting and, at the single cell level, by GFP tagging technology and immunocytochemistry, these approaches are not able to explain which process contributes to the fluctuations in the steady state levels of the proteins. New high-throughput technologies combining labeling of amino acids by stable isotopes and quantitative mass spectrometry to monitor protein turnover on a global scale were recently developed [1], [3] and applied also to plants [4]. Although these methods allow measuring simultaneously the abundance, synthesis, degradation and turnover of many proteins in a single experiment and in combination with cell fractionation even at a subcellular level [5], their sensitivity does not reach single cell resolution.

During the last decade, fluorescent protein techniques greatly speeded up cell biology research [6]–[8]. Unfortunately, fluorescence intensity as a measure of protein abundance provides information only about the net turnover rate of proteins. In this respect, the introduction of photoconvertible proteins that can shift their fluorescence emission from one wavelength to another has the potential to become an important tool for the estimation of different aspects of protein dynamics [7]–[12]. Several reports have appeared during the last years on the use of photoconvertible proteins for tracking proteins, subcellular structures and their interactions also in plants [13]–[18]. Dendra, a green-to-red photoconvertible protein tag was introduced by the Lukyanov’s laboratory [19] and the Dendra2 variant was advertized as a suitable tag to study protein dynamics since it is monomeric and photoconvertible by non-toxic blue light [19]–[21]. Dendra2 has been used to follow protein and cell structure dynamics in animals [20]–[30] and very recently to follow the movement of transcription factors in Arabidopsis roots [11] and to visualize PIN1 internalization after its transient expression in tobacco and Arabidopsis leaves [31]. In the present study, we have introduced Dendra2 as a tool to study protein turnover in the plasma membrane using PIN2 as a model.

Arabidopsis *PIN2* (*AtPIN2*, also known as *EIR1, AGR1* and *Wav6)* belongs to the PIN gene family whose members have been demonstrated to code auxin transporters in plants [32], [33]. As auxins are involved in divergent processes, many growth and developmental events are dependent on the steady-state of PIN proteins in the plasma membrane. PIN2 is expressed primarily in root apices and the protein is polarly localized in the plasma membrane of the root cortex and epidermis [34]. PIN2, similarly to other membrane proteins, is highly dynamic in the membrane and undergoes continuous internalization, recycling, degradation and membrane delivery. Many factors such as different plant growth regulators [35]–[39], stress [40], [41], light- dark exposure [42]–[44] and gravity [45], [46] may affect the localization and recycling patterns of PIN2. The internalization of PIN2 was shown to be dependent also on its post-translational modification [47] and proteasome function [45]. In previous studies the abundance of PIN2 in the membrane and its endocytosis have been mostly evaluated by GFP tagging technology in combination with genetic and pharmacological approaches. In order to track PIN2 relocalization in cells, Dhonukshe *et al*. [13] utilized EosFP, the most commonly applied green-to-red photoconvertible fluorescent protein. However, according to the authors, EosFP was inferior to GFP in term of sensitivity and in the highly fluorescent transgenic line PIN2-EosFP exhibited a less polarized distribution. Moreover, its expression was driven by the constitutive 35S promoter and PIN2 cDNA was utilized as coding sequence. Here we apply a more natural construct and demonstrate that by monitoring the green and red fluorescence emitted by the unconverted and photoconverted PIN2-Dendra2 in stable transformed plants it is possible to quantify directly, simultaneously and on a large-scale level the rate of PIN2 internalization and its delivery to the plasma membrane.

## Results and Discussion

### PIN2-Dendra2 is Functional and shows the Correct Localization Pattern in Transgenic Plants

**Figure 1:**
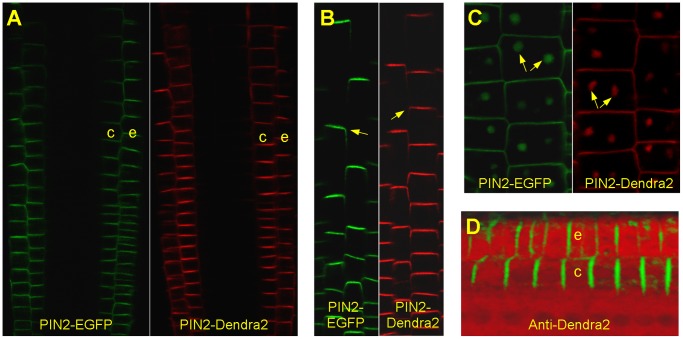
Localization pattern of PIN2-Dendra2 fusion protein. The roots of *PIN2-Dendra2* were photoconverted before imaging. (**A**) PIN2-Dendra2 when driven by the endogenous promoter is expressed in the root tip epidermis (e) and cortex (c) identically to PIN2-EGFP that was described previously [48]. (**B**) In both *PIN2-Dendra2 and PIN2-EGFP* transgenic lines, the fusion proteins localized polarly in shootwards transversal membranes (arrows). (**C**) PIN2-Dendra2 similarly to PIN2-EGFP accumulates in BFA bodies (arrows). (**D**) Expression and localization pattern of PIN2-Dendra2 was confirmed by immunohistochemistry using an anti-Dendra2 antibody (green) on chemically fixed and sectioned roots. Sections were counterstained by propidium iodide (red).

To create the *PIN2-Dendra2* construct we used the *PIN2* genomic DNA sequence including the endogenous promoter and regulatory sequence situated downstream the stop codon. We incorporated Dendra2 into the loop downstream of an alanine in position 403. In stable transformed plants the PIN2-Dendra2 fusion protein similarly to the commonly used PIN2-EGFP fusion [48], was exclusively expressed in the root epidermis and cortex (Figure 1 A), localized as a rule in transversal membranes at shootwards poles of epidermal cells (Figure 1 B) and accumulated in Brefeldin A (BFA) bodies (Figure 1 C). The localization pattern was confirmed by immunohistochemistry using a Dendra2-specific antibody (Figure 1 D).

Additionally, to confirm the functionality of PIN2-Dendra2 we performed experiments to rescue the gravity defects of *pin2*. We crossed *eir1-4,* the null-mutant allele of *PIN2*, showing an agravitropic phenotype [45] with the homozygous *PIN2-Dendra2* line and performed segregation analysis using the gravitropic assay as characterized previously [49]. Only 48 from 721 F2 progenies of self-pollinated double hemizygous F1 plants showed gravitropic defects. This means a segregation ratio (SR) of 14.1∶1 of gravitropic to agravitropic seedlings instead of a SR of 3∶1 expected providing that the mutation is not complemented by PIN2-Dendra2. When double hemizygous plants were crossed into *eir1-4/eir1-4,* from 111 F2 progeny seedlings only 29 were agravitropic (i.e. the SR was 2.8∶1 of gravitropic to agravitropic seedlings instead of the expected 1∶1 for functionless PIN2-Dendra2). No agravitropic seedling showed the expression of PIN2-Dendra2 when analyzed under an epifluorescence microscope. Finally, the progenies in both experiments showed the expected PIN2-Dendra2 positiveness/negativeness segregation ratios (∼3∶1 and ∼1∶1 respectively, not shown). Together, these data demonstrate that the *PIN2-Dendra2* transgenic line was able to complement the agravitropic phenotype of *eir1-4*. The identical localization to that described in previous studies using either PIN2 specific-antibodies or *PIN2-GFP* constructs [34], [44]–[46], [48] and the rescue experiments suggest the native behavior of the PIN2-Dendra2 fusion protein.

### The Cultivation of Seedlings on Microscope Slides is Suitable for Long Term Imaging by CLSM

For the current studies we cultivated seedlings on medium solidified with agar on microscope slides. Growth regulators and drugs were applied directly into the solid medium. The agar was trimmed at one end and seedlings were placed on the agar in a way that cotyledons lapped over the edge of the agar medium to avoid squashing by the cover glass. These slides were kept in Petri dishes with cultivation medium containing agar to protect slides and seedlings from desiccation. This simple cultivation system has several advantages: the straight root growth facilitates imaging of whole root tips, slides are easy to manipulate allowing uncomplicated gravitopism experiments and cover slips adhere well to the agar medium without the need for additional fixing before imaging. Most importantly, even after 24 h of cultivation and regular imaging no negative effects on growth and PIN2 signal pattern and turnover were observed provided that the cover slip was removed after every imaging (see below).

### Dendra2 Emission Spectra are pH-dependent

*In vitro* studies by Adam *et al.* [50] using Dendra2 expressed and isolated from *E. coli* suggest that both unconverted green and photoconverted red forms of this protein may appear in either anionic or neutral forms. All varieties showed a characteristic excitation and emission spectra and the ratio of anionic and neutral chromophores in solution were strictly pH-dependent. So far, however, these results have not been confirmed *in vivo*.

In initial experiments we carried out in *vivo* investigations on the emission spectra under different pH conditions. We analyzed root hairs of Arabidopsis seedlings expressing the Dendra2 protein under the control of the 35S promoter. As we found that after application of different buffers the cells did not change the emission spectra pattern probably because the cells kept their original pH, we had to treat seedlings in buffer solutions of appropriate pH for 5 min with the addition of 0.1% (w/v) formaldehyde to allow the diffusion of buffer components. After washing, the seedlings were kept in buffer of appropriate pH. Slightly fixed samples showed the fluorescence intensity still strong enough to allow spectra examination. Emission spectra were analyzed with the Meta detector of a Zeiss LSM-510 META CLSM (Figure S1). The data showed that also *in vivo* the emission spectra patterns of Dendra2 in the cytoplasm were strictly pH-dependent. The patterns in acidic conditions were quite complex, but at physiological conditions (around pH 7) there was only one dominant form of converted as well as unconverted Dendra2 both with single emission maxima when excited by either 488 nm or 543 nm laser lines (Figure S1). As the emission curves of unconverted and converted forms are not significantly overlapping, it was easy to set the appropriate laser, dichroic mirror and emission filter combinations to distinguish simultaneously both forms.

### Fluorescence Characteristics of Unconverted Dendra2 in vivo

In order to establish optimal conditions for live imaging, excitation and emission spectra of Dendra2 in different plant organs and cells of 35S::Dendra2 Arabidopsis seedlings were analyzed. The 35S promoter is widely used to drive gene expression in transgenic plants. Despite the variation in the efficiency of this promoter within organs and tissues [51], the promoter is constitutive in a manner that allows the analysis of Dendra2 fluorescence in different tissues. In addition, we analyzed the emission spectra of Dendra2 used as a tag attached to PIN2.

**Figure 2:**
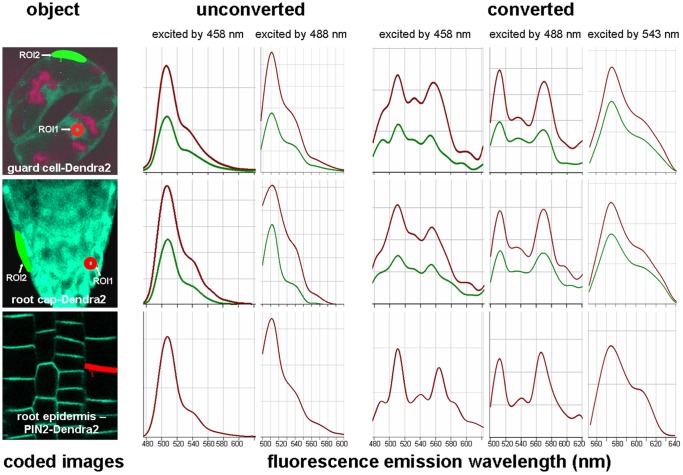
Representative spectra of Dendra2 before and after photoconversion. Dendra2 was expressed under the control of the 35S promoter and data for guard cells and root tips are presented. PIN2-Dendra2 driven by the endogenous promoter was analyzed in the membranes of root epidermis. Spectra were measured with a Zeiss LSM-510 Meta microscope before (labeled as unconverted) and after photoconversion (labeled as converted) for regions of interest (ROI) drawn in the coded images (left pictures in panel). Intact Dendra2 was analyzed in the nuclei (ROI1 drawn in the red color in the coded images) and in the cytoplasm (ROI2 drawn in green color in the coded images) of the guard cells and the cells of the root cap. Red lines in graphs represent the spectra emitted by nuclei, green lines represent the cytoplasm. The lower row in the panel shows the spectra emitted by the membrane-localized PIN2-Dendra2 (region drawn in red color in the coded image). The 458 nm excitation was combined with the HFT 458 beam splitter, the 488 nm excitation with the HFT 488 beam splitter and the 543 nm excitation with the HFT UV/488/543/633 beam splitter.

When either the 458 nm or 488 nm laser line was applied for excitation of unconverted intact Dendra2, the emission spectrum showed a single dominant peak in the green range, a shoulder in the yellow range and no peaks in the red range (Figure 2, labeled as unconverted). The patterns were very similar in both nucleus and cytoplasm and in all tissues of 35S::Dendra2 seedlings analyzed. An identical spectrum was emitted by the PIN2-Dendra2 fusion located in the plasma membrane (Figure 2, labeled as unconverted, Figure S2 A). When either 514 nm (not shown) or 543 nm laser line (Figure S2 C) was used for excitation, no emission signal was recorded in the red range. In summary, we found emission maxima at 506 nm and 511 nm when samples were excited by the 458 nm and 488 nm laser line respectively (Table S1).

### Photoconversion of Dendra2

According to Gurskaya *et al.* [19] Dendra2 can be converted by blue light. However using a variety of microscopes and filter settings we found it impossible to induce conversion of Dendra2 in our plants with blue light sources. We have also not succeeded to convert Dendra2 either with 458 or 488 nm lines of the argon laser of the confocal scanning microscope. Similar observations were also made by Wu *et al.* [11]. However, the failure of photoconversion of Dendra2 with blue light was in fact a great advantage for protein turnover studies (see below). First, the intensity of green signal in photoconversion experiments reflects the real protein synthesis rate, second, Dendra2 may be used as classical fluorescent protein in non-photoconverting experiments and third, there is no danger of incidental photoconversion of the green population during routine survey of samples in epifluorescence microscopy before and in the course of the experiments.

**Figure 3:**
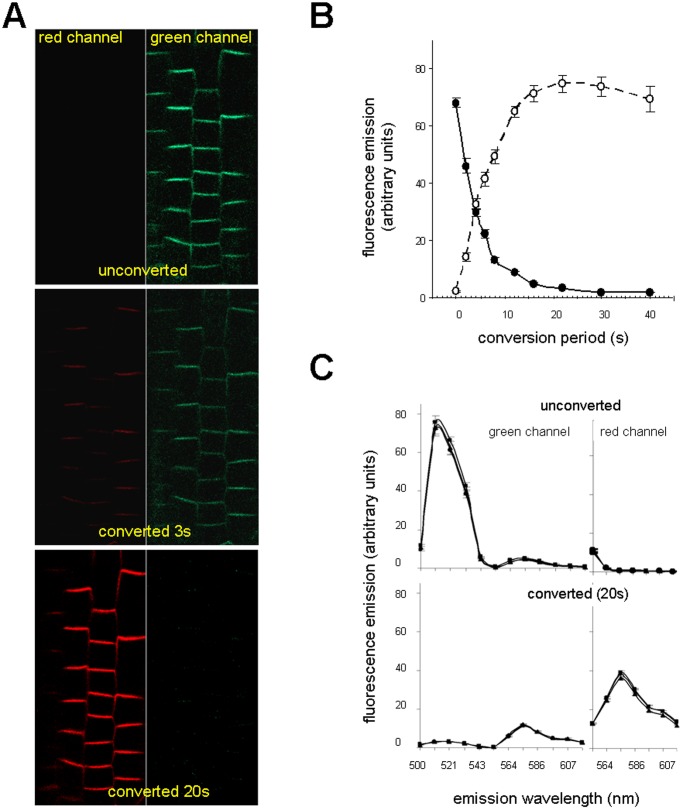
Time-dependent photoconversion of PIN2-Dendra2 fusion protein. (**A**) The same root was analyzed for green and red fluorescence before (time 0) and after 3 s and 20 s conversion. (**B**) Efficiency of photoconversion depends on the time of illumination with blue-violet light. Green and red fluorescence signals were collected and the intensities of 150 transversal membranes within 5 roots were assessed with the ImageJ software. The interrupted line with open circles represents changes in red signal intensities, the solid line with closed circles represents decreasing in green signal intensities, bars represent SE. (**C**) Emission spectra of green and red signals of unconverted and photoconverted membranes were analyzed in three roots (represented by three lines in the graph). The HFT UV/488/543/633 was used as a beam splitter. Each point on the graph with SE bar represents the mean of 30 transversal membranes.

In further experiments we investigated filter sets with an excitation band path around 400 nm (Nicon V-2, Leica D, Leica B/G/R) and we discovered that both free Dendra2 and Dendra2 tags were effectively converted within a relative short time. All subsequent conversions were carried out with a Leica DMR microscope equipped with a 100 W Mercury short arc lamp in combination with the Leica filter set D (excitation filter BP355–425, emission filter LP 470). Using a 40× objective and fully opened aperture we could illuminate and convert Dendra2 uniformly in the whole root segment where PIN2 expression occurs. Images captured in the green and red channels before conversion, and 3 s and 20 s after conversion by blue-violet light are presented in Figure 3 A. The conversion efficiency is strictly dependent on the duration of illumination (Figures 3 B and S3). Although a decrease in the green and an increase in the red signal intensity become apparent after a few seconds of illumination, it takes about 20 s of induction to reach maximal red emission intensity (Figures 3 B and S3). Some weak residual green emission remained in the membrane even after prolonged photoconversion (Figures 3 C, S2 B and S3). Compared to the original signal intensity, this residual fluorescence was down to 4% after 20 s and 2% after 60 s photoconversion. As violet irradiation may be harmful to the plants [52], samples were routinely converted for 15 s.

Prolonged illumination resulted in a decrease of the red signal of the converted Dendra2 form (Figures 3 B and S3). This may be caused either by partial photobleaching of the red Dendra2 or by a second photoconversion of the red Dendra2 into a super-red variety with even more red-shifted absorption and emission spectra as documented in *in vitro* conditions [50]. After prolonged photoconversion we also observed an additional emission peak that may correspond to the super red variety (Figure S3). We also verified that 15 s illumination had no significant inhibitory effect on root growth and gravitropism of *PIN2-Dendra2* seedling roots. After photoconversion seedlings were placed in a vertical position and root growth checked after 24 h. Control values (2523±292 µm) and treated root values (2402±280 µm) were according to the Student’s t-test (n = 30) statistically not different. In the gravitropism assay the position of slides was changed from vertical to horizontal after photoconversion and the curvature of root tips checked after 3 and 5 h. Differences in root growth directions were not statistically significant between control and blue-violet light-treated roots as evaluated by Student’s t-test (33.2±1.7° vs. 32.4±1.7° after 3 h and 42.2±1.5° vs. 41.3±1.6° after 5 h, values show means±standard errors of the mean, n = 30).

### Fluorescence Properties of Photoconverted Dendra2

Free Dendra2 and Dendra2 tag showed identical emissions when excited by a 543 nm laser line with a single maximum between 575.5 and 576 nm (Figure 2, labeled as converted, Figures 3 C, S2 D and S3, Table S1). This corresponds to the anionic red form of Dendra2 as characterized *in vitro* [50]. As mentioned above even after a prolonged conversion period we still observed some weak emission signal in the green range when samples were excited by either 458 or 488 nm laser lines. In comparison with unconverted samples, the emission patterns of converted samples were more complex. After excitation with the 488 nm laser line this emission spectrum showed three peaks situated between 512 and 513 nm, between 530 and 540 nm and between 565 and 570 nm (Figure 2, labeled as converted, Figure S2 B, Table S1). Compared to the unconverted form, the peak in the green range had a slight shift towards longer wavelength (Table S1). When photoconverted samples were excited by the 458 nm laser line, a shoulder emerged also in the blue area around 490 nm (Figure 2, labeled as converted). An emission curve in the red range with a peak around 560 nm corresponds to the anionic Dendra2 red form as characterized previously [50]. The spectral pattern with peaks in blue, green and yellow range strongly resembles the one emitted *in vitro* by the neutral Dendra2 red form [50] which according to the authors is a dominant variety in acidic conditions. We found levels of this form of Dendra2 to be very low in the plant cytoplasm, nucleus and plasma membrane which may be due to the relatively high pH in these compartments. We could not check the red spectra of intact Dendra2 *in vivo* under acidic conditions e.g. in vacuole, as intact Dendra2 was not targeted to this compartment. However, recent studies on PIN2 demonstrated that this protein is targeted to vacuoles for degradation under dark conditions [42]–[44]. As expected we observed partial redistribution of PIN2-Dendra2 into provacuole and vacuole-like structures after moving seedlings to darkness for 2 h (Figure S4 A). We checked the spectra emitted by these structures after photoconversion and found them to be different from that emitted by membrane PIN2-Dendra2 (Figure S4 B). The emission profile indicates the neutral form of red Dendra2 which seems to be the exclusive form in the acidic vacuole. These results suggest that Dendra2 may be used as a tag to detect simultaneously proteins in the vacuole and in the cytoplasm and membrane.

### Live Imaging in Protein Turnover Experiments

**Figure 4:**
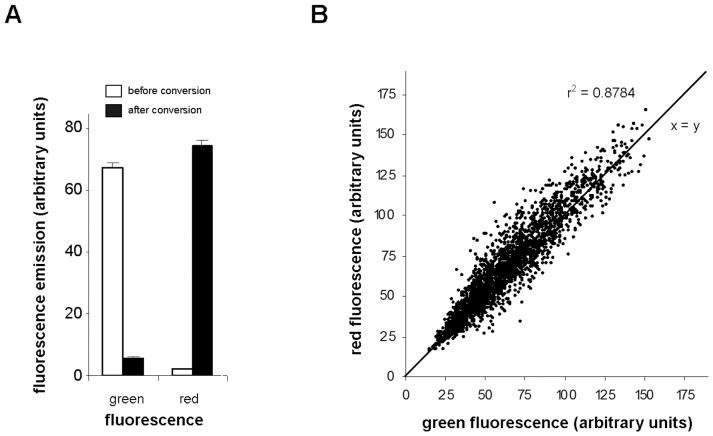
Values of green and red signal intensities in transversal plasma membranes containing PIN2-Dendra2. (**A**) Means of green and red membrane fluorescence intensities examined before and after photoconversion in arbitrary unites. The graph summarize the results obtained in the course of the whole study (with 330 roots 20 to 25 membranes per root were analyzed). (**B**) Values of green signal intensities emitted by the membranes before photoconversion were plotted against the values of red signal intensities emitted by the same membranes after 15 s’ photoconversion. The graph shows the results from a total of 2500 membranes from 100 roots used in the course of study.

Based on the spectral data of Dendra2 forms we adjusted the parameters for life-cell imaging of plasma membrane PIN2 dynamics. According to our in *vivo* data and *in vitro* studies [50], only one green form of Dendra2 is present in the unconverted state at physiological conditions. This form we excited with a 488 nm laser line and the signal was detected with a 505 to 530 nm band-pass filter. As we outlined above, the proportion of the neutral red form of Dendra2 *in vivo* seems to be negligible in comparison with the anionic red form, therefore we adjusted image capturing for the anionic red Dendra2 form. In our experiments we used a 543 nm laser line for excitation and a 565 to 615 nm band pass filter for detection of emission. Gain and offset parameters of the LSM detectors in the experiments on PIN2-Dendra2 turnover were set in such a way that the green signal of unconverted membranes and the red signal of converted membranes gave approximately the same values of around 70 arbitrary units of the 0 to 256 scale of the 8-bit color depth mode. Data on green and red fluorescence intensities obtained by imaging before and after conversion in course of the experiments are given in arbitrary units in Figure 4 A. After photoconversion the intensity of the green signal in the plasma membrane decreased 12.46±0.26 times (mean and standard error) and the intensity of red signal increased 73.15±6.05 times (around 7 000 membranes within 330 roots were analyzed in the course of study). These results show an approximately 900-fold increase in red-to-green fluorescence ratio. The conversion was very uniform through the whole root tip and was not significantly affected by the signal strength. Plotting the green and red signal intensities of individual membranes measured before and after conversion (2500 transverse membranes from 100 roots were analyzed) shows that the conversion pattern follows an almost linear regression model (Figure 4 B, P-values = 6.26×10^−16^).

### PIN2 Turnover in the Plasma Membrane

imilarly to other membrane proteins PINs are internalized by endocytosis and either targeted for degradation or recycled through endosomes back to the plasma membrane [32], [33]. Proteins are also *de novo* synthesized, then processed and delivered to the plasma membrane by exocytosis. We supposed that time-lapse imaging in photoconverting experiments combined with measurement of red and green signal intensities emitted by the PIN2-Dendra2 recombinant protein by laser confocal microscopy may allow to quantify protein internalization and delivery to the plasma membrane. Because the photoconversion of Dendra2 is an irreversible process [20]–[22] a decline of red signal strength in the plasma membrane over time should be proportional to the internalization and subsequent degradation of the PIN2-Dendra2 fusion protein. On the other hand the increase of green signal intensity in the plasma membranes after photoconversion should reflect the delivery of *de novo* synthesized PIN2-Dendra2 to the membrane. The protein turnover eventually should lead to the replacement of the original red population of the recombinant proteins by the green form. Moreover unconverted Dendra2 was found to be photostable and not converted by laser light under the conditions used for routine imaging therefore we were able to use unconverted Dendra2 as classical FP for an overall estimation of fusion protein abundance in the plasma membrane. Since absolute values of green signal intensities before conversion and absolute values of red signal intensities after conversion are approximately the same (see above), the sum of green and red intensities at particular time points should be approximately equal to the intensity of signal at the same time point in parallel non-photoconverting experiments.

**Figure 5:**
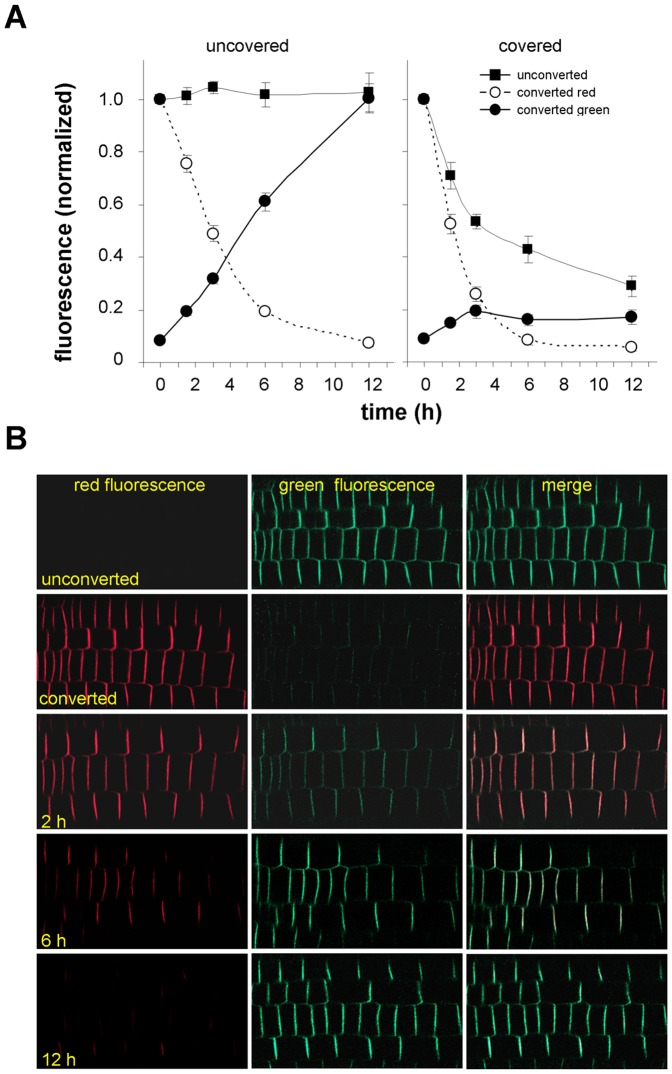
Turnover of PIN2 in the membranes of root tips under normal and anaerobic conditions. (**A**) The cover slips were either removed after every imaging (labeled as uncovered) or roots were permanently covered by cover slips (anaerobic conditions). Converted red in the labeling means red signal intensity in transversal membranes after photoconversion; converted green in the labeling means green signal intensity after photoconversion; unconverted in the labeling means green signal in the membrane without photoconversion. (**B**) Images of the same roots taken in green and red channels characterizing the decrease of red signal intensity and increase of green signal intensity in membranes within 12 h after photoconversion.

Surprisingly, in a series of preliminary experiments when we kept seedling roots permanently enclosed with cover slips we were not able to confirm the above outlined scenario. Green fluorescence intensity of unconverted samples in covered roots strongly declined over time (Figure 5 A, labeled as covered), however, when the cover slip was removed after every imaging, fluorescence intensity was kept on steady state level (Figure5 A, labeled as uncovered). Photoconversion experiments showed that the reduction in fluorescence intensity in covered roots was actually caused by lack of recovery of the green fluorescence (Figure 5 A, labeled as uncovered). When the cover slip was removed after every imaging the recovery of green signal intensity in photoconverting experiments was accomplished within 12 h (Figure 5 A, labeled as uncovered and 5 B). Hence, 12 h incubation was selected mostly for the subsequent experiments.

**Figure 6:**
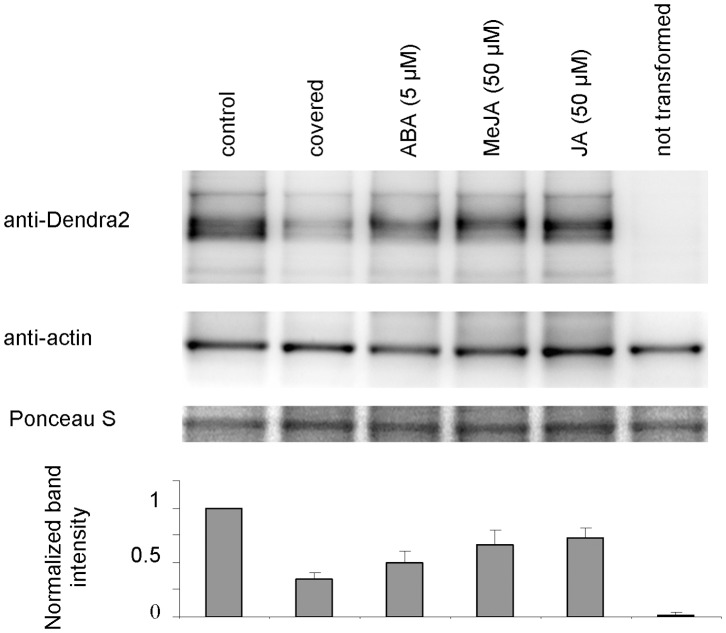
Western blot analysis of PIN2-Dendra2 expression. For Western blot analysis seedlings were grown and treated as described for microscopy and roots were collected after 12 h’ treatment for protein extraction. The lane marked as ‘covered’ represents extracts from seedlings whose roots grew for 12 h under anaerobic conditions under the cover slip. Control (untreated) samples, samples of untransformed seedlings and samples treated with ABA and jasmonates (MeJA and JA) were kept uncovered during the experiment. The blot was probed by a Dendra2 specific antibody (upper row), stripped and re-probed with an actin specific antibody (middle row). The lower row shows the membrane after Ponceau S staining. Bands intensities were quantified using ImageJ. The values obtained for Dendra2 were divided by the values for actin for that sample, normalized to the control and graphed. Columns in the graph represent the means of three blots, bars represent SD.

Deficit in green signal recovery may be a consequence of PIN2-Dendra2 membrane delivery shortage. However, since FPs maturation is oxidation-dependent [53], this failure may also be caused by the suppression of the Dendra2 chromophore maturation under anaerobic conditions. To address this important question, we performed a similar experiment with covered *PIN2-EGFP* [48], the construct commonly used in previous studies on PIN2, and with *35S::Dendra2* seedlings. When seedling roots were permanently enclosed by cover slips we observed a strong decrease in fluorescence intensity of membranes in *PIN2-EGFP* seedlings after 8 and 12 h cultivation, however we recorded only a slight diminishing of fluorescence signal intensity in the cytoplasm of root cells in *35S::Dendra2* seedlings (Figure S5 A). Additionally we carried out western blotting analysis of *PIN2-Dendra2* roots using an antibody against Dendra2 and demonstrated a dramatic reduction in the total amount of fusion protein in seedlings with covered roots (Figure 6). Importantly, we also observed a reduction in root growth by 31.33% and 42.24% within 24 and 48 h, respectively when compared to seedlings with uncovered roots. Root growth inhibition may be caused by the reduction of PIN2 abundance in the membrane. Taking together, anaerobic conditions strongly inhibit PIN2 membrane delivery most likely as a consequence of synthesis inhibition. The immersion of seedlings in solution with different drugs is a common practice in studies with PIN proteins. Attention should be paid to possible side effects of anaerobic conditions during prolonged treatments.

Two approaches were used to estimate the half-life of the PIN2-Dendra2 fusion protein. The first calculation was based on the mean time for 50% reduction of red signal intensity in the membrane taking the intensity immediately after conversion as 100%. In the second approach the mean time for recovery of 50% green signal intensity was measured, here putting the intensity before photoconversion at 100%. From the curves for the red signal in controls a half-life of 3 h 38 min±8 min (mean±SE) was calculated, which equals a PIN2 turnover of almost 14% per 1 h. In case of the green signal, 50% of the original green signal intensity was reached within 4 h 37 min±8 min, indicating that 10.8% of the membrane localized PIN2 is exchanged every hour by *de novo* synthesized protein. Internalization and delivery rate should be approximately equal to maintain the steady stay of protein in the membrane at the same level. The somewhat longer residence time of PIN2 in the membrane estimated from the green emission recovery can be explained by a slow maturation of the Dendra2 chromophore. If slow enough, a subset of the PIN2-Dendra2 molecules is transported to the membrane when its chromophore maturation is not completed and they do not have absorption/emission in the blue/green spectral range.

High-throughput technologies monitoring protein turnover on a global scale in yeast [54], human [5] and Arabidopsis [4] have revealed an incredible variation in turnover rate among proteins. In Arabidopsis cell suspension cultures the exchange of glutathione peroxidase 6 was almost forty times slower than the turnover of ACC oxidase [4]. In HeLa cells approximately 60% of the proteins have a half-life of 15 to 25 h. Only about 10% proteins have a half-life less than 10 h [5]. In yeast, from more that fifty highly abundant proteins analyzed, the highest exchange rate of about 9% per h was assessed for methionine synthase [54]. Taking together, when we compare the half-life of PIN2 with the available data we can conclude that PIN2 is short-living protein indeed.

### PIN2 Turnover in the Plasma Membrane is Affected by Many Factors

**Figure 7:**
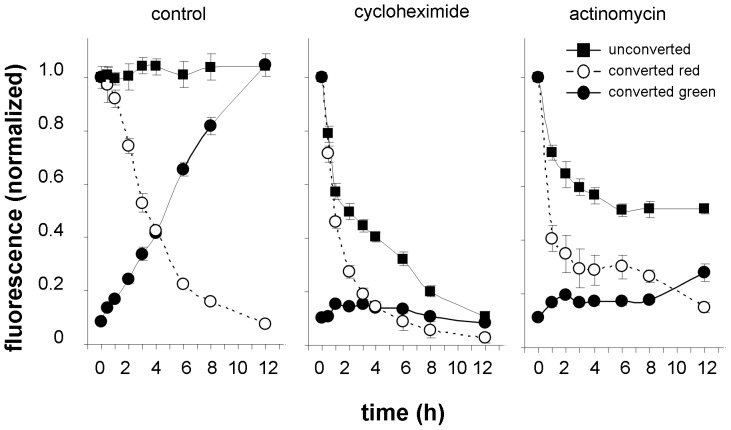
Cycloheximide (50 µM) and actinomycin A (50 µg ml^−1^) block PIN2 membrane delivery and accelerates PIN2 internalization. The differences between control and samples treated by antibiotics were statistically highly significant (p≤0.001) when analyzed by the two-way ANOVA test in all events i.e. green and red signal intensities in photoconverting experiments and also in green signal intensity of unconverted samples. Bonferroni’s post test showed highly statistically significant differences (p≤0.001) between control and cycloheximide-treated seedlings at all time points for red signal intensities in photoconverting experiments and for green signal intensity in experiments with unconverted samples. Significant differences were observed in green signal intensity in photoconverting experiments at every time point from 2 to 12 h (for all p≤0.001). In the experiments with actinomycin A statistically significant differences between control and treated samples in red signal intensity were observed at every time point from 1 to 3 h (p≤0.001) and at time point 4 h (p≤0.05), in green signal intensity at time point 3 h (p≤0.01) and at every time point from 4 to 12 h (p≤0.001) and in green signal intensity of unconverted samples at all time points (p≤0.001). For others, the differences were statistically not significant.

The suitability of the FP photoconverting approach to study protein turnover was analyzed in a series of experiments under conditions affecting either the synthesis, delivery or internalization of PIN2. In the presence of actinomycin A and cycloheximide, antibiotics known to block transcription and translation, respectively, a rapid decrease of green signal intensity in the plasma membrane was observed in non-photoconverting experiments (Figure 7). Both antibiotics inhibited green PIN2-Dendra2 plasma membrane pool recovery in roots after photoconverting as a consequence of inhibition of the protein synthesis pathway. In the case of cycloheximide, effects became evident within 1 h after application, in the case of actinomycin A effects were observed within 2 h (Figure 7). Actinomycin A has an overall stronger effect than cycloheximide when both antibiotics were applied at commonly used concentrations. Interestingly, the antibiotics also sped up the withdrawing of the red signal from membranes implying an accelerated PIN2-Dendra2 internalization after antibiotic treatments. This indication should be considered in experiments with these widely used inhibitors. As these antibiotics block the translation and transcription nonspecifically, the increase in PIN2 internalization may be a consequence of reduced synthesis of some other protein(s) required for the stabilization of PIN2 at the membrane.

**Figure 8:**
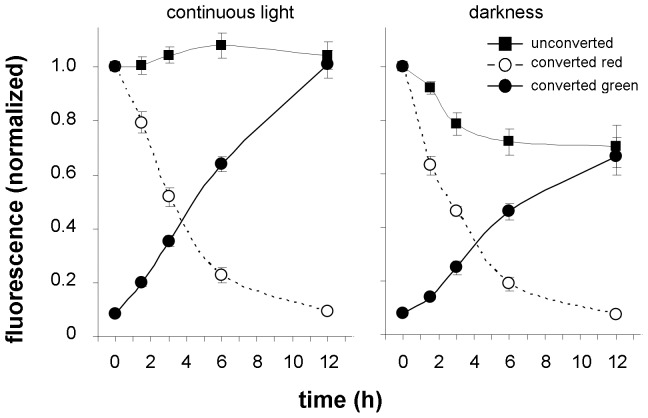
Darkness treatment diminishes PIN2 abundance in the membrane mostly by suppressing its delivery. Relocation of seedlings from the light to darkness significantly affected green and red signal intensities in photoconverting experiments and also green signal intensity of unconverted samples (in all cases p≤0.001). In photoconverting experiments, differences in green signal were detected at time point 6 h (p≤0.05) and 12 h (p≤0.001), in red signal at time point 1.5 h (p≤0.001) and in experiments without photoconversion at every time point from 3 to 12 h (p≤0.001). For others, the differences were statistically not significant.

Physical factors can also affect PIN2 dynamics. A relocation of PIN2 from the membrane to the vacuoles occurs when plants are transferred into darkness [42]–[44]. By investigating the effect of transferring seedlings into the dark, we observed a decrease in signal intensity with unconverted Dendra2 samples (Figure 8) comparable to the results published by Laxmi *et al.* [42]. Photoconversion approach shows only a slight reduction in red signal intensity but a significant inhibition of green signal recovery when compared with samples growing in continuous light (Figure 8).

**Figure 9:**
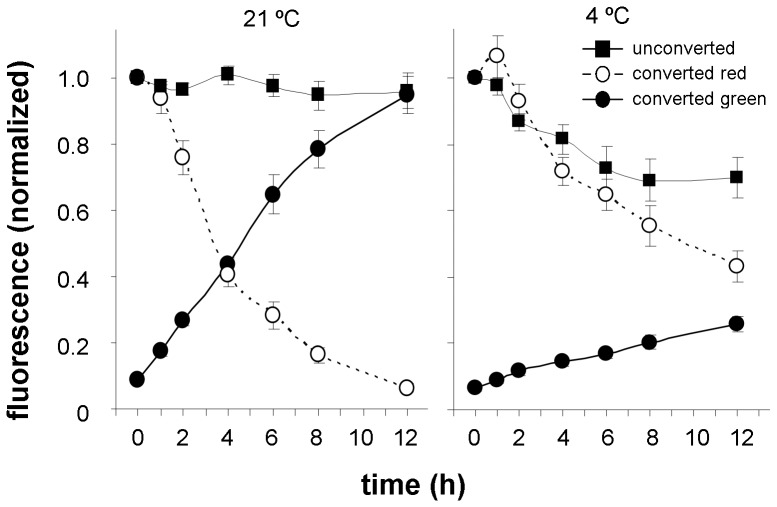
Low temperature treatment inhibits the internalization and membrane delivery of PIN2. Differences between samples kept at 21°C and 4°C were statistically highly significant in all cases (p≤0.001) i.e. red signal intensities and green signal intensities in photoconverting experiments and green signal intensities in non-photoconverting experiments. In photoconverting experiments differences in green signal intensity were observed at every time point from 2 to 12 h (p≤0.001), in red signal intensity at time point 1 h (p≤0.001) and at every time point from 2 to 12 h (p≤0.001) and in experiments without photoconversion at time point 4 h (p≤0.01) and at time points from 6 and 12 h (p≤0.001).

**Figure 10:**
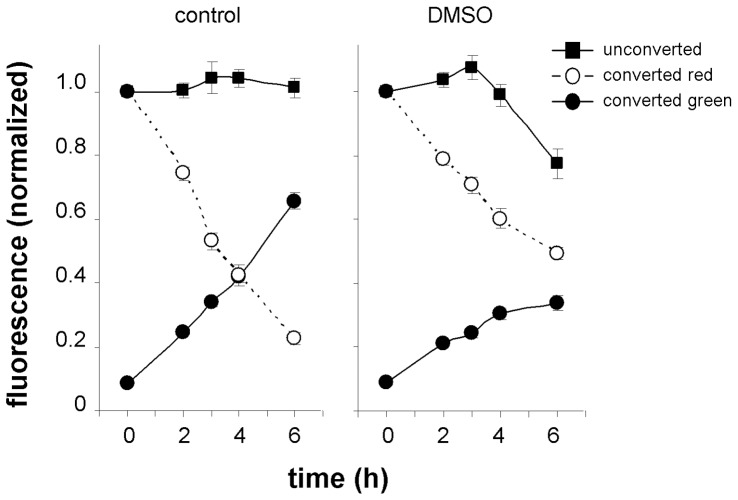
DMSO affects PIN2 internalization and membrane pool recovery. A statistically highly significant effect (p≤0.001) of 3% (v/v) DMSO applied in the medium was observed in all events i.e. green and red signal intensities in photoconverting experiments and also in green signal intensity in non-photoconverting experiments. Differences between control and DMSO-treated samples in experiments with unconverted samples were observed at time point 6 h (p≤0.001), in photoconverting experiments for green signal intensity at time point 4 h (p≤0.001) and 6 h (p≤0.001) and for red signal intensity at time point 3 (p≤0.01), 4 and 6 h (both p≤0.001).

Low temperature as a stress factor generally inhibits metabolism and growth processes. Moreover low temperature, similarly to DMSO, rigidifies membranes [55] and the decrease in membrane fluidity potentially may reduce protein internalization by endocytosis. In our non-photoconverting experiments incubation of seedlings at low temperature (4°C) resulted in an overall decrease of PIN2 abundance in the plasma membrane in (Figure 9). For the assessment of PIN2 internalization and membrane pool recovery by photoconversion experiments we analyzed the red and green signal dynamics and found that low temperature treatments strongly inhibited both processes (Figure 9). A 50% reduction of red signal intensity in the membrane when compared with that observed immediately after conversion was reached after approximately 10 h indicating at least a three-fold increase in half-life turnover of PIN2 in the plasma membrane. A repression of PIN2 trafficking in the cold has also been recognized by Shibasaki *et al.* [41]. Furthermore we observed similar inhibitory effects on PIN2 internalization and increased half-life in the presence of 3% (v/v) DMSO (Figure 10). The results suggest that low temperature and DMSO may act in a similar way i.e. by increasing the rigidity of the plasma membrane thus hampering the internalization of membrane proteins by endocytosis. Green signal recovery was also significantly inhibited by DMSO probably as a consequence of general toxic effects of this substance on protein synthesis.

**Figure 11:**
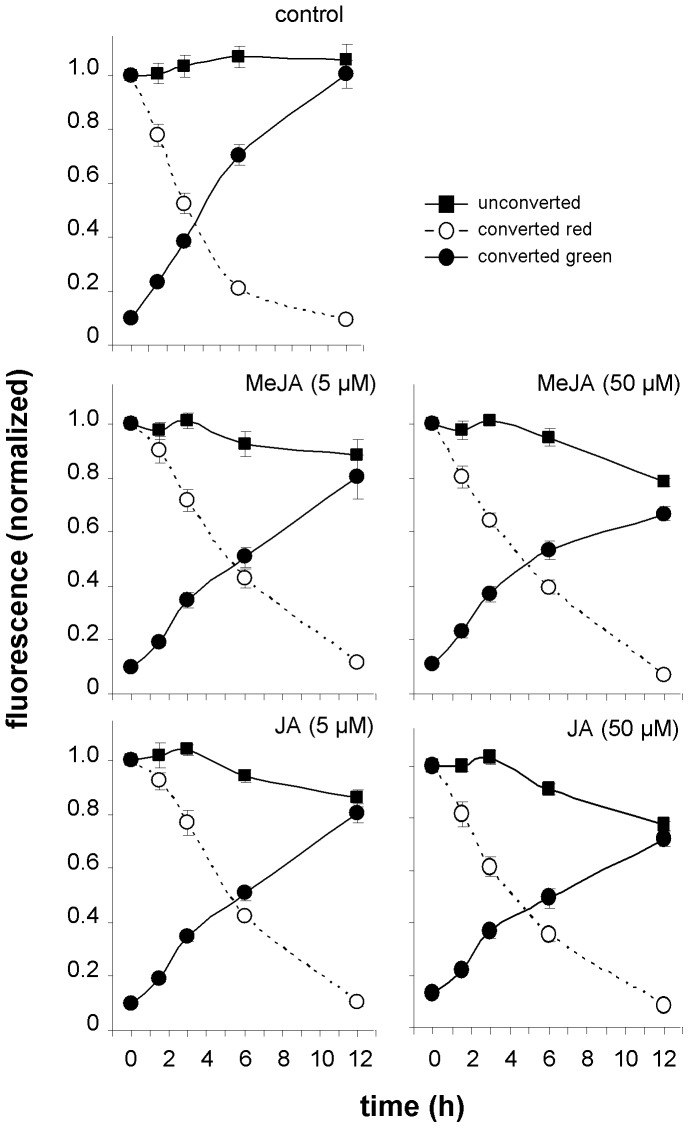
Jasmonates influence PIN2 plasma membrane dynamic. Both JA and MeJA at the concentrations 5 µM and 50 µM affect green signal intensity in transversal plasma membranes of unconverted samples (p≤0.01 for 5 µM JA and 5 µM MeJA, p≤0.001 for 50 µM JA and 50 µM MeJA,). Differences were significant at time point 6 h (p≤0.05) and 12 h (p≤0.001) for 5 µM JA, at time point 6 h (p≤0.001) and 12 h (p≤0.001) for 50 µM JA, at time point 6 h (p≤0.05) and 12 h (p≤0.01) for 5 µM MeJA, and at time point 6 h (p≤0.05) and 12 h (p≤0.001) for 50 µM MeJA. For others differences were statistically not significant. Both jasmonates at the concentrations examined slowed-down the disappearance of red signals from the membrane (labeled as converted red; for each jasmonate and concentration p≤0.001) For both jasmonates used at a concentration of 5 µM differences were significant at time point 1.5 h (p≤0.01), 3 h (p≤0.001) and 6 h (p≤0.001), for 50 µM JA at time point 3 h (p≤0.05) and 6 h (p≤0.001), for 50 µM MeJA at time point 3 h (p≤0.05) and 6 h (p≤0.05). For others, the differences were statistically not significant. Both jasmonates at the concentrations examined delayed membrane green signal recovery (labeled as converted green; for each jasmonate and concentration p≤0.001)). Differences were significant at time point 6 h (p≤0.001) and 12 h (p≤0.001) for 5 µM JA, at time point 6 h (p≤0.01) and 12 h (p≤0.01) for 5 µM MeJA, at time point 6 h (p≤0.001) and 12 h (p≤0.001) for 50 µM JA and 50 µM MeJA. For others, the differences were statistically not significant.

Finally we examine the influence of abscissic acid (ABA) and two jasmonates i.e. jasmonic acid (JA) and methyl jasmonate (MeJA) on PIIN2 turnover. These plant hormones are involved in regulating various aspects of plant growth, development ands responses to different stresses [56], [57]. Recently Sun *et al.* [37] showed by BFA application that MeJA pretreatment at concentrations of 5 µM slowed down PIN2 endocytosis in Arabidopsis through its interaction with the auxin pathway. At higher concentrations (50 µM) MeJA reduced the abundance of PIN2 in the membrane by an auxin signaling-independent mechanism. Complete results of our experiments on jasmonates are shown in Figure 11. With unconverted samples we were able to confirm the reduction of PIN2 abundance in the plasma membrane of seedling roots grown on the medium with 50 µM MeJA and by a less extend also on medium with 5 µM MeJA. A similar effect was observed when JA, a close relative to MeJA, was applied at the same concentrations. These results were supported by western blot analysis using a Dendra2-specific antibody that showed a significant decrease in the total amount of PIN2-Dendra2 fusion protein (Figure 6). Furthermore *PIN2-EGFP* transgenic line [48] gave very similar results to that obtained with our construct (Figure S5 B). By a photoconversion approach we found that both jasmonates at the concentration of 5 and 50 µM significantly hamper the disappearance of red fluorescence from membranes. This suggests that the internalization of PIN2 is inhibited by jasmonates. Furthermore, in photoconverting experiments we observed the significant inhibition of membrane green signal intensity recovery in PIN2-Dendra2 seedling roots after prolonged treatment with both jasmonates. This effect was dose dependent.

**Figure 12:**
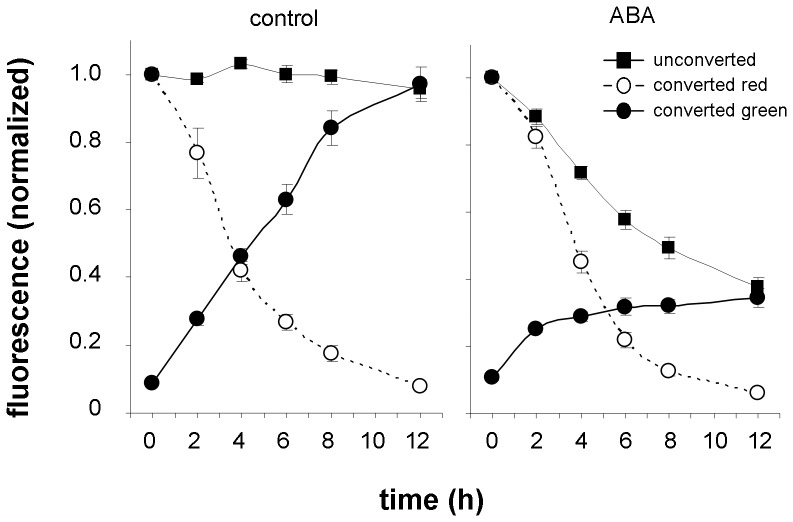
ABA inhibits PIN2 membrane delivery. ABA (5 µM) exhibited no effect on red signal intensity in photoconverting experiments, and affected green signal intensity in both photoconverting and non-photoconverting experiments (for both p≤0.001). The differences between control and treated samples in non-photoconverting experiments were significant at every time point from 2 to 12 h (for each p≤0.001), the differences in photoconverting experiments were significant at time point 1 h (p≤0.05) and at every time point from 2 to 12 h (for all p≤0.001).

A significant reduction of fluoresce signal intensity in membranes of unconverted roots was found after 5 µM ABA application (Figure 12). This result was again confirmed by western blotting (Figure 6) and in the experiment with *PIN2-EGFP* transgenic line (Figure S5 B). In photoconverting experiments we observed strong suppression of PIN2-Dendra2 green pool recovery, however, no effect on PIN2 internalization was recorded (Figure 12). Interestingly public available transcriptome data (https://www.genevestigator.com) suggest that both ABA and JA at high concentrations (10 µM and 100, respectively) downregulate significantly PIN2 transcription. The failure in membrane PIN2 pool renewal in the experiments with jasmonates and ABA may be a consequence of decreased protein abundance in the cells due to the downregulation of PIN2 gene expression.

In conclusion, the results presented in this study make clear that the Dendra2 photoconverting fluorescent protein technology offers several unique advantages to study protein turnover. Most importantly it allows to quantify simultaneously the removal rate of protein from membrane and membrane protein delivery rate of newly synthesized protein. Moreover, in parallel experiments the non-ptotoconverted Dendra 2 probes may be used as classical tags. The method is noninvasive, it allows to investigate protein turnover at the level of single cell resolution, the procedure is very precise, accurate, highly reproducible and relatively simple. A similar approach may be applied to study the turnover of other plasma membrane proteins and proteins localized in distinct subcellular compartments.

## Materials and Methods

### Vector Construction and Plant Transformation

To construct the *p35S::Dendra2* vector, *Dendra2* cDNA sequence was amplified by PCR with *D2-F1* and *D2-R1* primers (Invitrogen, Karlsruhe, Germany; sequences of all primers are listed in Table S2) by Elongase (Invitrogen, Karlsruhe, Germany) from a *Gateway®Dendra2-At-N* entry clone (Evrogen, Moscow, Russia) and inserted between *Cla*I and *Xba*I sites on *pAMPAT-MSC* (GenBank: AY436765.1) vector. To generate the binary vector encompassing *pPIN2::PIN2-5′ end::Dendra2::PIN2-3′ end::pAPIN2* cassette (abbreviated as *PIN2-Dendra2* in the text), upstream 4048 bp long DNA fragment containing the 1811 bp-long promoter region sequence and the 5′ end of the *PIN2* gene was amplified by PCR from genomic DNA with *PIN2-F1* and *PIN2-R1* oligos, downstream 1877 bp long DNA sequence encompassing 3′ end of *PIN2* gene and a 328 bp long DNA fragment located downstream stop codon containing *pA* site of the gene was amplified by PCR from genomic DNA by *PIN2-F2* and *PIN2-R2* oligos, *Dendra2* was PCR amplified from a *Gateway®Dendra2-At-N* entry clone (Evrogen) by *D2-F2* and *D2-R2* oligos. Fragments were inserted sequentially into the *pAMPAT-MSC* vector between *Asc*I and *Not*1 sites. Restriction and other DNA processing enzymes were from New England Biolabs (Frankfurt, Germany). Sequence and reading frames were proved by sequencing analysis. Constructs were transformed into *Arabidopsis thaliana* ecotype Columbia (Col-0) using *Agrobacterium tumefaciens GV3101( pMP90RK)* [58] with the floral-dip method [59]. Selection of homozygous plants was performed by growing seedlings on medium supplemented with 7.5 mg/l phosphinothricin (PPT, Duchefa, Haarlem, The Netherland). Resistant seedlings were screened under an epifluorescence microscope for the expression of Dendra2. The *PIN2-EGFP* line was a generous gift of Prof. Ben Scheres (Utrecht University) and was described previously [48].

### Plant Material, Cultivation Conditions, Media and Chemicals

We selected the transgenic line carrying a single T-DNA insertion exhibiting an intermediate level of PIN2-Dendra2 expression and obtained homozygous seeds. Homozygous plants were used for the experiments and for crossing into plant homozygous for *pin2*. The experiment seeds were surface sterilized by 1% (w/v) sodium hypochlorite and germinated in Petri dishes with solid MSAR medium [60]. Dishes were kept in vertical position in a growth chamber at 21°C under continuous light (100 µmol m^−2^ s^−1^). All chemicals were from Sigma-Aldrich (St. Louis, USA) unless otherwise stated.

The functionality of the construct was verified by rescue experiments. Homozygous *PIN2-Dendra2* plants were used as pollen donors for crossing into plant *eir1-4* allele homozygous for *pin2* (45). F1 hemizygous plants were either allowed to self pollinate or were crossed back into *eir1-4.* Gravitropic response test with the F2 progeny seedlings was performed as described previously [49]. Shortly, seedlings were growing alongside the medium in vertically-placed Petri dishes for five days and than the plates were turned so that roots were oriented horizontally. Segregation analysis of seedlings on the basis of root growth reorientation was performed after two days.

### Photoconversion and Confocal Microscopy

Conversion of PIN-Dendra2 in transgenic roots was performed with a Leica DMR upright microscope (Leica Microsystems, Wetzlar, Germany) equipped with a mercury lamp of 100W, a 40× Fluotar objective and a filter cube D (excitation BP355–425/emission LP 470) applying 15 s illumination. PIN2 turnover was quantitatively analyzed in root meristem epidermal cells. Longitudinal median optical sections through the root epidermis layer adjacent to the objective were captured with a Zeiss LSM-510 META inverted confocal laser scanning microscope (Carl Zeiss Jena GmbH, Germany). Recordings of 320×550 pixels were acquired with 8-bit color depth using a 20×/0.75 Plan Apochromat objective, pinhole setting at 140 µm, 4× line averaging and pixel dwell time of 2.56 µs. For green signal capture, samples were excited with a 488-nm line of argon laser and the signal was collected with a 505 to 530 nm band-pass filter. For red signal, samples were excited by the 543-nm line of the HeNe laser and fluorescence was collected with a 565 to 615 nm band-pass. Red and green signals were acquired in multi-track mode and for both channels HFT UV/488/543/633 was used as main dichroic mirror. Identical settings were kept to collect images in the course of the whole study. For emission spectra analysis, different excitation laser lines in combination with appropriate main dichroic mirrors were applied and fluorescence emissions were collected with the spectral Meta detector.

### Western Blotting and Immunohistology

For western blotting, either 5 days-old seedlings or excised roots were ground in RIPA lysis buffer (150 mM NaCl, 50 mM Tris pH7.4, 1 mM EDTA pH 8.0, 1% (v/v) Triton-X100, 0.1% (w/v) SDS (sodium dodecyl sulfate), 0.8% (w/v) DOC (sodium deoxycholate), protease inhibitor cocktail (P9599, Sigma-Aldrich, St. Louis, USA), 1 mM PMSF (phenylmethylsulfonyl fluoride). Extracts containing 30 µg protein were blotted onto Immobilon-P membrane (Millipore Corporation Bedford, USA) and probed with anti-Dendra2 antibody (Evrogen, Moscow, Russia, dilution 1∶5000) dissolved in TBST buffer (25 mM Tris-HCl pH 7.5, 150 mM NaCl, 0.1% (v/v) Tween 20) supplemented with 3% (w/v) skim milk for 1 h. After washing 3 times for 5 min with TBST buffer, membranes were incubated with 0.2 µg ml^−1^ HRP-conjugated goat anti-rabbit IgG (Bio-Rad, Munich, Germany) dissolved in 3% (w/v) skim milk in TBST for 1 h. After washing three times for 10 min in TBST, signals were visualized by Pierce ECL Western Blotting substrate (Thermo Scientific, Rockford, USA).

For immunohistology sections were prepared and probed as described previously [51]. Primary anti-Dendra2 antibody was diluted 1∶200, a FITC-conjugated goat anti-rabbit IgG secondary antibody (Sigma-Aldrich, St. Louis, USA) was diluted 1∶100. Samples were counterstained with propidium iodide (1 µg ml^−1^) and examined in a Zeiss LSM-510 META inverted confocal laser scanning microscope (Carl Zeiss Jena GmbH, Germany). Green signals were excited with a 488-nm laser line and captured with a 505 to 530 nm band-pass filter, red signals were excited with a 543-nm laser line and captured with a 565 to 615 nm band-pass filter.

### Experiment Design and Data Evaluation

Before starting treatments, seedlings were transferred onto the fresh MSAR solid medium for 12 h. In photoconverting experiments four to five seedlings were transferred on microscope slides coated with 1.5 ml solid cultivation medium, covered by cover slip and imaged as mentioned above. Roots were then photoconverted for 15 s (see above) and again imaged. After imaging seedlings were immediately moved onto new microscope slides covered by solid medium supplied with the effector compound; control seedlings were placed on medium without effector compounds. Parallel non-photoconverting experiments were performed with seedling without photoconversion. During the course of the experiment microscope slides containing seedlings were placed on solid cultivation medium in Petri dishes (9 cm diameter) and kept under light and at 21°C (if not otherwise stated) until the next measurement. The Petri dishes were stored vertically so that seedlings stood upright. Before imaging, a cover slip was placed on seedlings and removed again after imaging. Roots were re-imaged several times within 12 h under the same microscope settings. Time points in the graphs represent the period of treatment either by the chemical compound or the physical factor. Means as parameters for green and red signal intensities of transversal epidermal membranes were recorded by Image J software (NIH, Bethesda, USA) using the straight line selection mode with five pixel line width. Twenty membranes were investigated at every time point in every four to five roots used for the experiment. Experiments were repeated independently three times. Despite we have used a homozygous transgenic line we observed some variability in signal intensity among individual seedlings therefore results on green and red signal intensities in experiments on PIN2 turnover were not expressed in arbitrary units but as relative values. In single roots the means of green signal intensities at every time point during the experiment were normalized to the mean of green signal intensities emitted by the membranes of the same root before conversion, i.e. means of green fluorescence intensities at each time point were divided by the mean of fluorescence intensity recorded before conversion (in panels labeled as converted green and in graph presented as solid lines with filled circles). Time points 0 represent relative green signal intensities recorded immediately after conversion e.g. just prior to the treatment application. Analogically in single roots the means of membrane red signal intensities at every time point during the experiment were related to the mean red fluorescence intensity measured soon after conversion (time point 0), e.g. shortly before the treatment application (in panels labeled as converted red and in graphs presented as interrupted lines with open circles). Values of the time points 0 were set to 1. In parallel non-photoconverting experiments means of green signal intensities at every time point during the experiment were normalized to the mean of green signal intensities emitted by the membranes of the same root before application of treatment (in panels labeled as unconverted and in graphs presented as solid line with filled squares). Values of the time points 0 were set to 1.Graphs were prepared using the Microsoft Excel (Microsoft, Redmont, USA) package. Points displayed in the graphs represent the means of 12 to15 roots, bars correspond to standard errors of the means (SE). Statistical significances between treated and control samples were determined by two-way ANOVA test and differences between means of control and treated samples at single time points were identified by a Bonferroni’s post hoc test using the GraphPad Prism 5.00 Software (San Diego, USA). Images were processed with Adobe Photoshop CS2 (Adobe Systems, Mountain View, USA) and Microsoft Publisher (Microsoft, Redmont, USA) softwares.

## Supporting Information

Figure S1 **Influence of pH on the emission spectra of Dendra2.** Dendra2 was expressed under the 35S promoter and spectra were analyzed in the cytoplasm of root hairs by a Zeiss LSM-510 Meta microscope using the lambda scan mode. Fluorescence intensities were normalized automatically by the instrument software (LSM 510, rel 3.2). Seedlings with unconverted and converted roots were slightly fixed for 5 min with 0.1% (w/v) freshly prepared formaldehyde in buffer of indicated pH values. Buffers for pH 3 to 7 were prepared mixing 50 mM citric acid and 100 mM potassium hydrogen phosphate, buffers for pH 8 to 11 were prepared mixing 100 mM sodium carbonate and 100 mM potassium dihydrogen phosphate.
(TIF)

Figure S2 **Lambda image galleries for PIN2-Dendra2 fusion protein.** Images were taken with the Meta detector in lambda mode in the range of 500 to 650 nm before (A and C) and after conversion (B and D). 488 nm’s laser line was combined with the HFT 488 beam splitter and 543 nm laser line was combined with the HFT 488/543/633 beam splitter.
(TIF)

Figure S3 **Patterns of emission spectra of PIN2-Dendra2 after different periods of photoconversion.** 488 nm’s excitation was combined with the HFT 488 main beam splitter and 543 nm’s excitation was combined with the HFT UV/488/543/633 main beam splitter.
(TIF)

Figure S4 **Emission spectra of PIN2-Dendra2 localized in the membrane and in the vacuole.** After moving seedlings from light to darkness, PIN2-Dendra2 in root meristematic cells was partially re-localized from the plasma membrane (arrowheads in coded image in A) to small vacuoles (arrows in A). Spectra emitted by the PIN2-Dendra2 fusion of photoconverted samples after 458 nm laser excitation in combination with the HFT 458 main beam splitter are shown in B. Red line represents the spectra emitted by the membrane-located PIN2-Dendra2 (the area encircled by red line in coded image in A), green line represents the spectra of vacuole-located PIN2-Dendra2 (area enclosed in green circle line in coded image in A)
(TIF)

Figure S5 **Unconverted** ***PIN2-Dendra2*** **roots show identical response to anaerobic and plant growth regulators treatments as** ***PIN2-EGFP*** **roots.** (A) In both PIN2 transgenic lines the signal intensity in transversal membranes decreased dramatically when roots were permanently enclosed by cover glass. In transgenic lines that expressed free Dendra2 under the 35S promoter only a slight decrease of cytoplasm signal intensity in both, root cup and root meristematic cells, was noticed. (B) ABA (5 µM) and jasmonates (50 µM) show a similar effect on fluorescence diminishing from the membrane in both PIN2 transgenic lines.
(TIF)

Table S1 **Emission maxima of Dendra2.** Material, excitation conditions and beam splitters were as described in Figure 1. Peak maxima were determined at least in 50 ROIs and means with standard deviations are presented.
(TIF)

Table S2 **Sequences of primers used for PCR.**
(TIF)
