# Supplementary figures and images for: PIN2 Turnover in Arabidopsis Root Epidermal Cells Explored by the Photoconvertible Protein Dendra2
Source: PLoS One. 2013 Apr 18;8(4):e61403. doi: 10.1371/journal.pone.0061403 (PMC3630207; doi:10.1371/journal.pone.0061403)

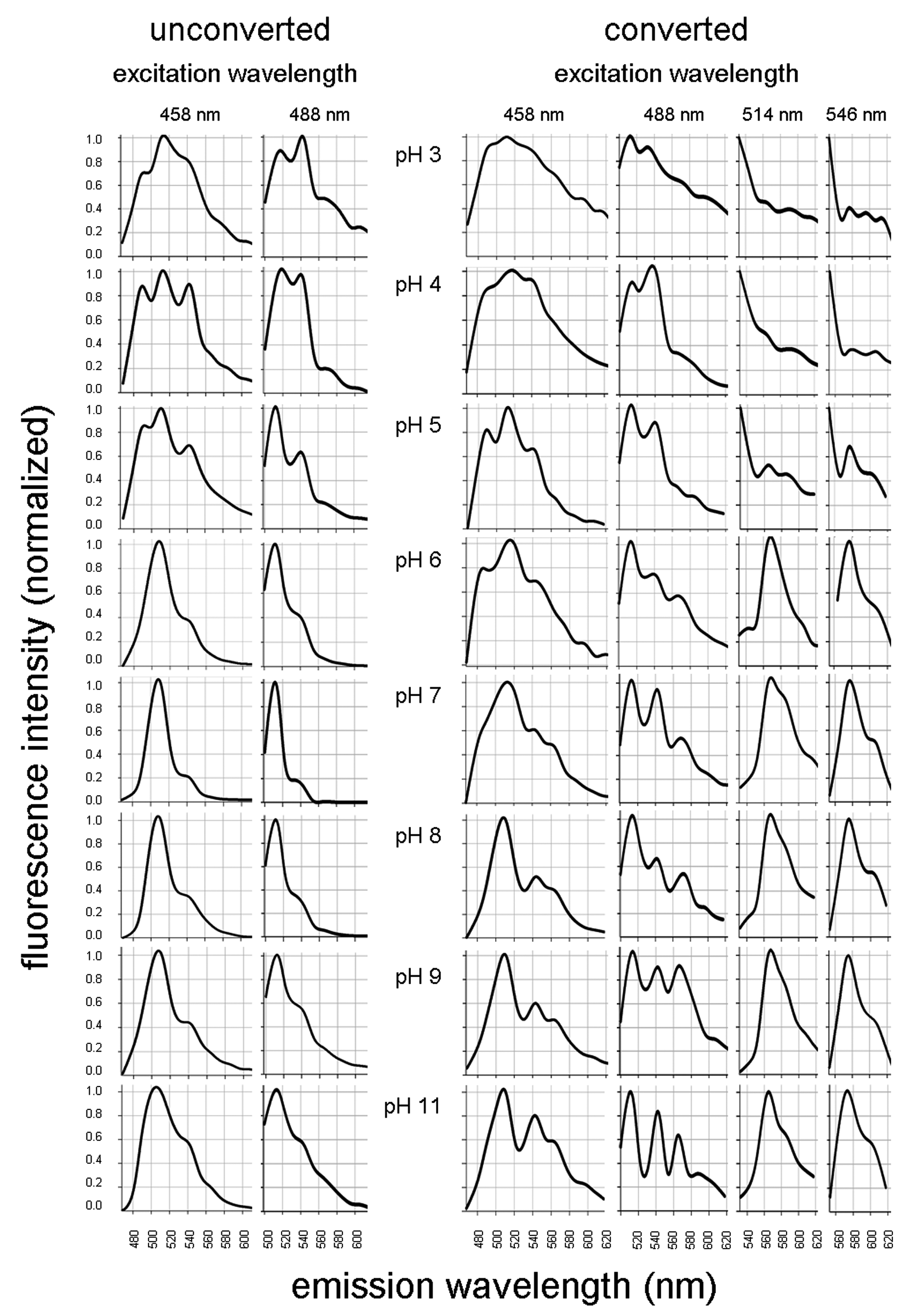

Supplement: Figure S1 — Influence of pH on the emission spectra of Dendra2. Dendra2 was expressed under the 35S promoter and spectra were analyzed in the cytoplasm of root hairs by a Zeiss LSM-510 Meta microscope using the lambda scan mode. Fluorescence intensities were normalized automatically by the instrument software (LSM 510, rel 3.2). Seedlings with unconverted and converted roots were slightly fixed for 5 min with 0.1% (w/v) freshly prepared formaldehyde in buffer of indicated pH values. Buffers for pH 3 to 7 were prepared mixing 50 mM citric acid and 100 mM potassium hydrogen phosphate, buffers for pH 8 to 11 were prepared mixing 100 mM sodium carbonate and 100 mM potassium dihydrogen phosphate. (TIF) [file pone.0061403.s001.tif]

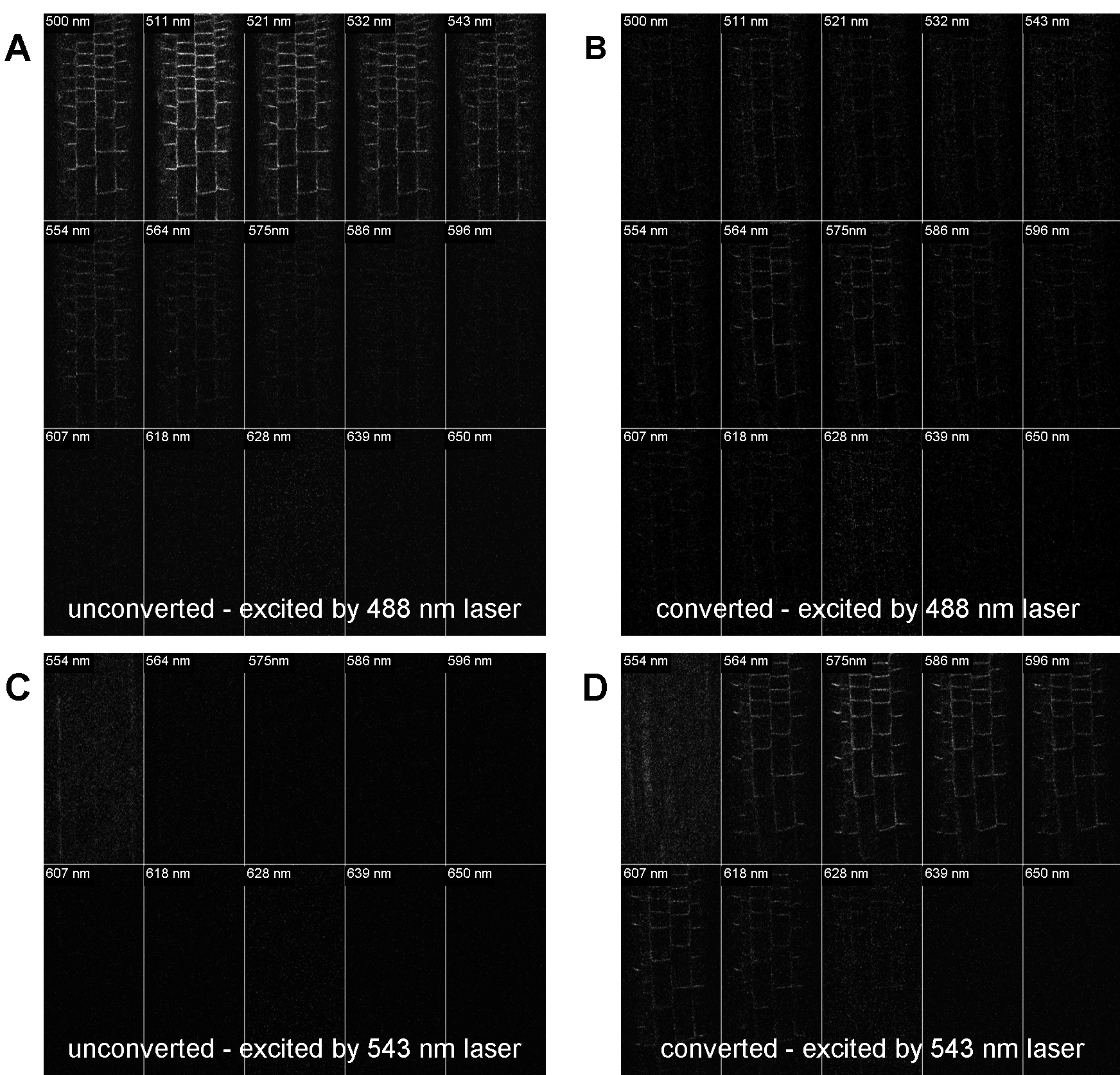

Supplement: Figure S2 — Lambda image galleries for PIN2-Dendra2 fusion protein. Images were taken with the Meta detector in lambda mode in the range of 500 to 650 nm before (A and C) and after conversion (B and D). 488 nm’s laser line was combined with the HFT 488 beam splitter and 543 nm laser line was combined with the HFT 488/543/633 beam splitter. (TIF) [file pone.0061403.s002.tif]

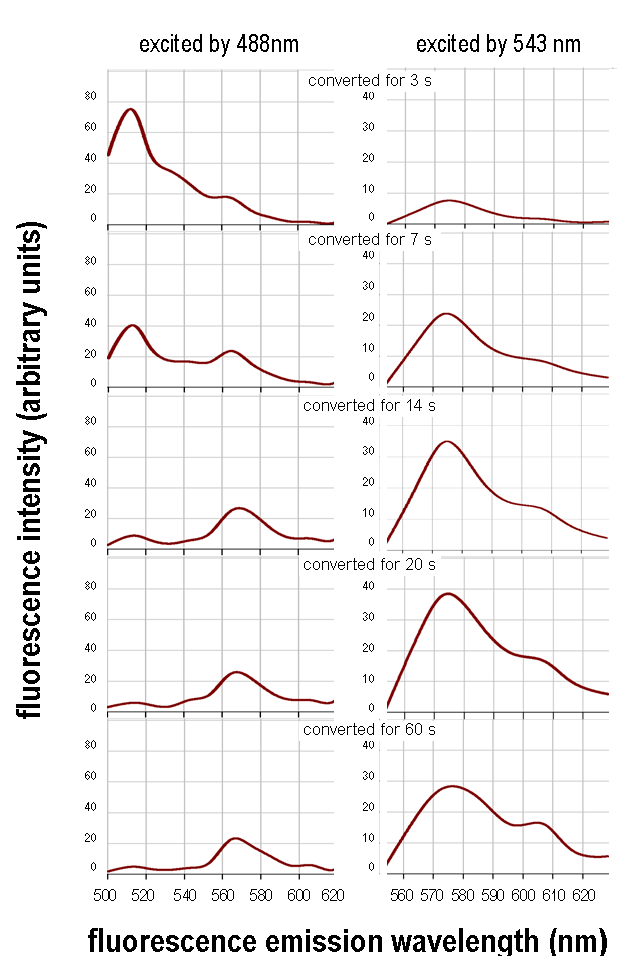

Supplement: Figure S3 — Patterns of emission spectra of PIN2-Dendra2 after different periods of photoconversion. 488 nm’s excitation was combined with the HFT 488 main beam splitter and 543 nm’s excitation was combined with the HFT UV/488/543/633 main beam splitter. (TIF) [file pone.0061403.s003.tif]

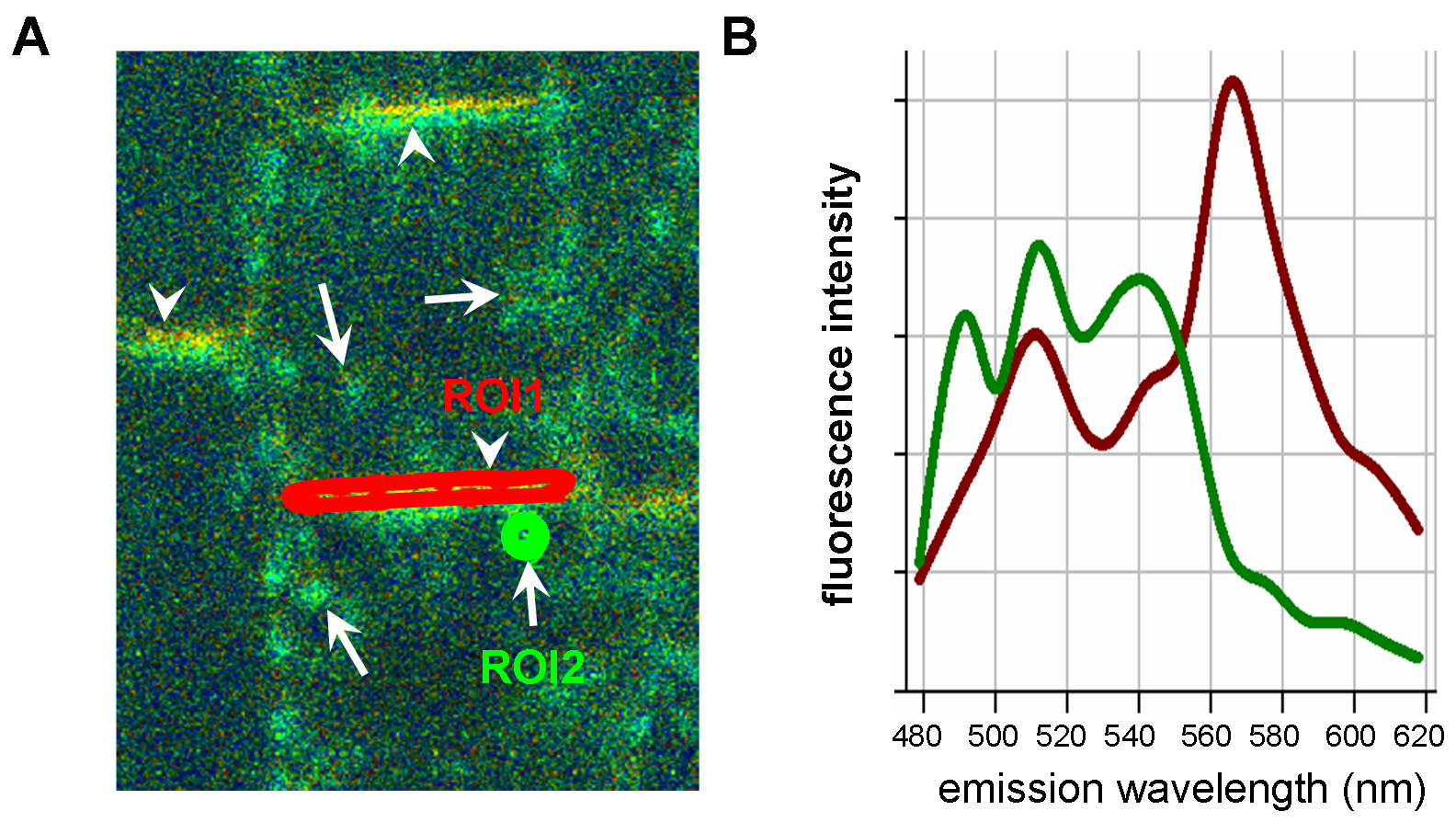

Supplement: Figure S4 — Emission spectra of PIN2-Dendra2 localized in the membrane and in the vacuole. After moving seedlings from light to darkness, PIN2-Dendra2 in root meristematic cells was partially re-localized from the plasma membrane (arrowheads in coded image in A) to small vacuoles (arrows in A). Spectra emitted by the PIN2-Dendra2 fusion of photoconverted samples after 458 nm laser excitation in combination with the HFT 458 main beam splitter are shown in B. Red line represents the spectra emitted by the membrane-located PIN2-Dendra2 (the area encircled by red line in coded image in A), green line represents the spectra of vacuole-located PIN2-Dendra2 (area enclosed in green circle line in coded image in A) (TIF) [file pone.0061403.s004.tif]

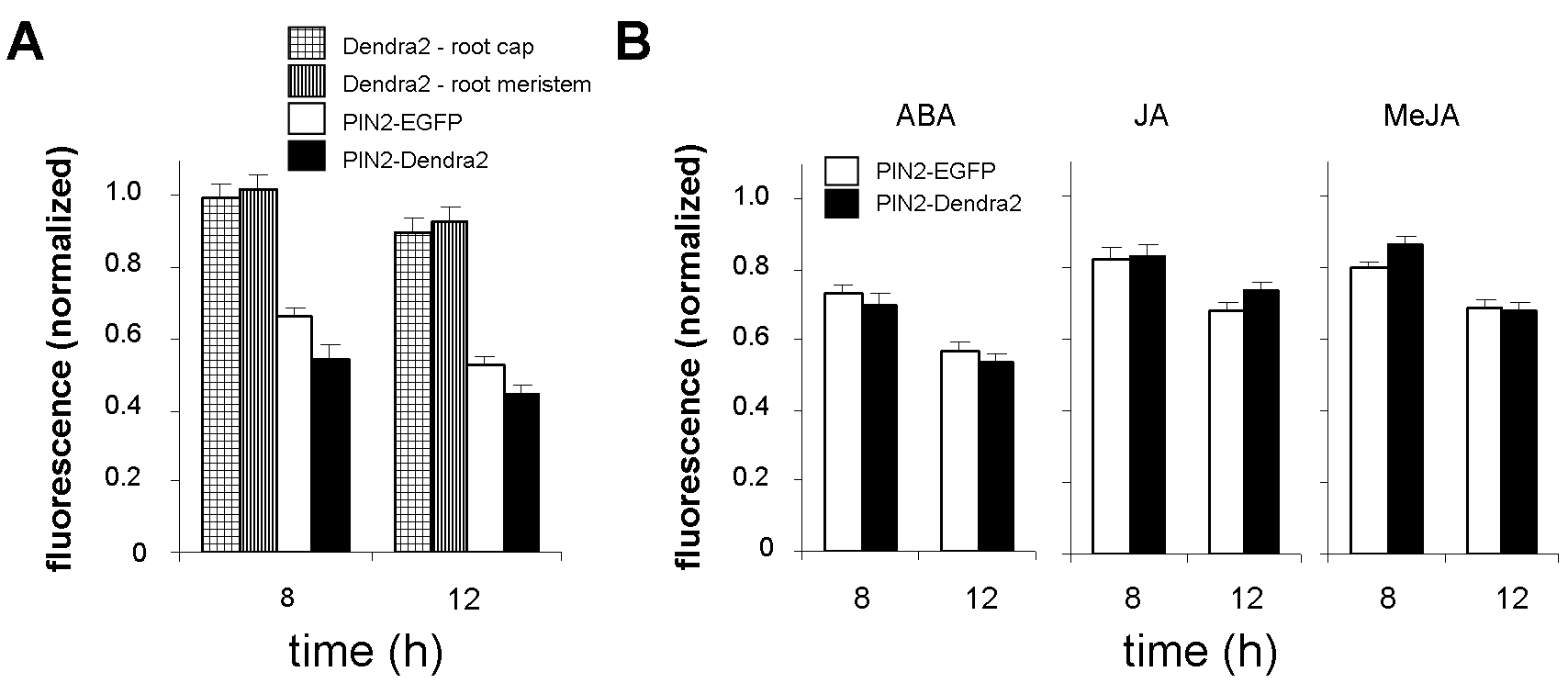

Supplement: Figure S5 — Unconverted PIN2-Dendra2 roots show identical response to anaerobic and plant growth regulators treatments as PIN2-EGFP roots. (A) In both PIN2 transgenic lines the signal intensity in transversal membranes decreased dramatically when roots were permanently enclosed by cover glass. In transgenic lines that expressed free Dendra2 under the 35S promoter only a slight decrease of cytoplasm signal intensity in both, root cup and root meristematic cells, was noticed. (B) ABA (5 µM) and jasmonates (50 µM) show a similar effect on fluorescence diminishing from the membrane in both PIN2 transgenic lines. (TIF) [file pone.0061403.s005.tif]

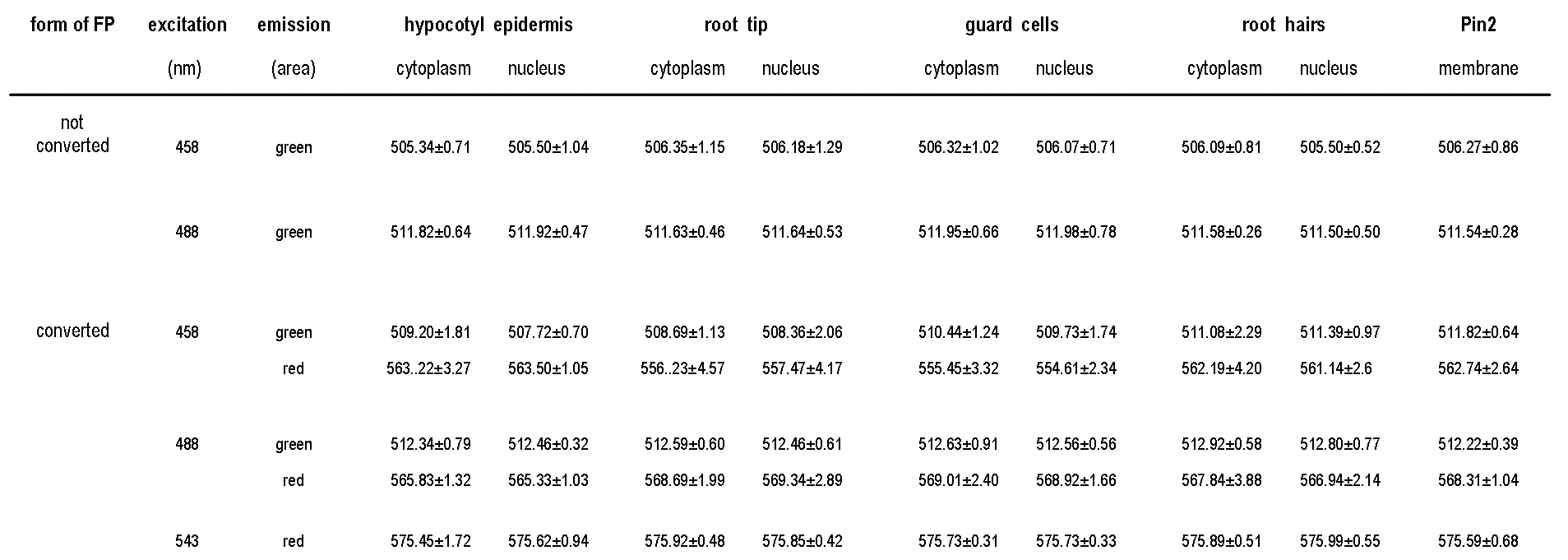

Supplement: Table S1 — Emission maxima of Dendra2. Material, excitation conditions and beam splitters were as described in Figure 1. Peak maxima were determined at least in 50 ROIs and means with standard deviations are presented. (TIF) [file pone.0061403.s006.tif]

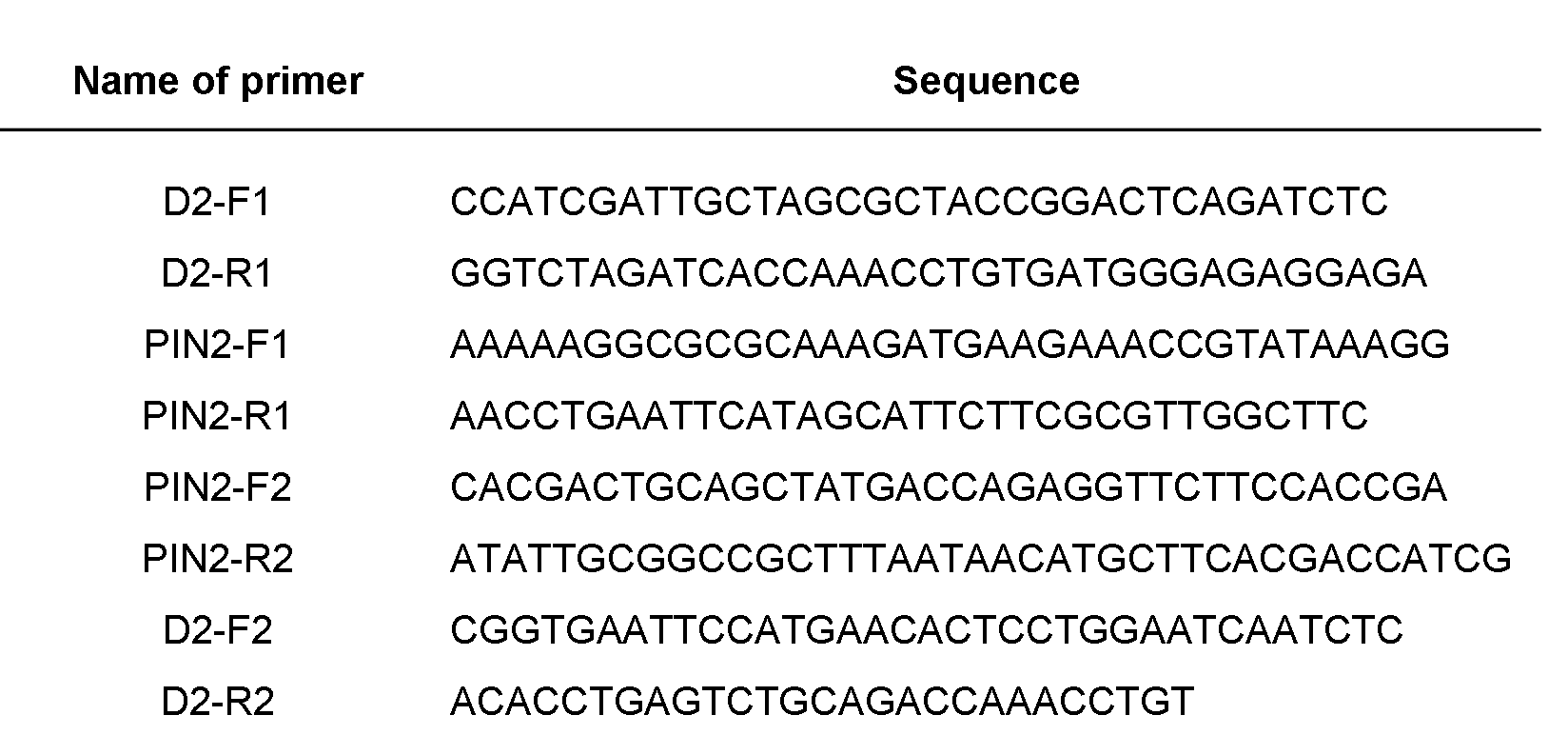

Supplement: Table S2 — Sequences of primers used for PCR. (TIF) [file pone.0061403.s007.tif]
